# Supplementary material for: Asynchronous magnetic resonance elastography: Shear wave speed reconstruction using noise correlation of incoherent waves
Source: Magn Reson Med. 2022 Oct 27;89(3):990–1001. doi: 10.1002/mrm.29502 (PMC9792433; doi:10.1002/mrm.29502)
Supplement: Supplementary file 1 — DATA S1 MATLAB code used to generate the numerical simulations of Figure 1 [file MRM-89-990-s002.zip › k-Wave/helpfiles/interpftn.html]

interpftn :: Functions (k-Wave)


# interpftn

Resample data using Fourier interpolation.

## Syntax

```
y = interpftn(x, sz)
```

## Description

`interpftn` resamples an N-D matrix to the size given in `sz` using Fourier interpolation.

## Inputs

|  |  |
| --- | --- |
| `x` | matrix to interpolate |
| `sz` | new size |

## Outputs

|  |  |
| --- | --- |
| `y` | resampled matrix |

## See Also

`interpft`
